# Manipulating mitochondrial electron flow: a novel approach to enhance tumor immunogenicity

**DOI:** 10.1186/s43556-024-00171-5

**Published:** 2024-03-20

**Authors:** Junyu Wang, Anren Zhang, Min Wu, Shugang Qin

**Affiliations:** 1 Department of Rehabilitation Medicine, Shanghai Fourth People’s Hospital Affiliated to Tongji University School of Medicine, Shanghai, 200434 China; 2 Wenzhou Institute, University of Chinese Academy of Sciences, Wenzhou, 325000 Zhejiang China; 3 The Oujiang Laboratory Zhejiang Lab for Regenerative Medicine Vision and Brain Health, Wenzhou, Zhejiang China; 4 Department of Critical Care Medicine, Frontiers Science Center for Disease-related Molecular Network, State Key Laboratory of Biotherapy and Cancer Center, West China Hospital, Sichuan University, Chengdu, China

A recent study published in *Science* by *Kailash* et al. revealed a novel approach for enhancing tumor immunogenicity by manipulating mitochondrial electron flow [1]. The mitochondrial tricarboxylic acid (TCA) cycle and electron transport chain (ETC) are recognized as pivotal in offering the metabolic adaptability crucial for the proliferation and advancement of cancer. Specifically, the continuous electron flow, mediated by mitochondrial respiratory chain complex I (CI) and CII, is crucial for tumor growth [2, 3]. Nevertheless, the distinct roles of CI and CII in initiating electron flow were not well-defined previously. The study by *Kailash* and colleagues concentrated on elucidating the separate contributions of CI and CII to tumor development, metabolic processes, and immunogenic characteristics.

To evaluating the roles of CI and CII in tumor proliferation and the anti-tumor immune response, the research team generated CI (sgNdufa1) or CII (sgSdha or sgSdhc) deficient YUMM1.7 (Braf^V600E^/Pten^−/−^/Cdkn2a^−/−^) through targeted genetic engineering. These modified cells were then implanted into homozygous wild-type C57BL/6 mice. The findings demonstrated that while defects in CI did not significantly affect tumor growth, impairments in CII notably reduced tumor progression in melanoma. This decline in growth coincided with numerous noteworthy observations: enhanced infiltration of CD8^+^ T cells, augmented secretion of IFN-γ^+^ and GZMB^+^, and a significant elevation in the expression of major histocompatibility complex class I (MHC-I). These results suggest that functional defects in CII lead to robust anti-tumor immune responses, primarily through enhanced antigen presentation.

*Kailash* and colleagues studied how CII defects enhance tumor antigen presentation. The researchers observed that the application of 3-nitropropionic acid (3-NPA) enhanced MHC-I expression across multiple mouse cell lines (YUMM1.7, 4 T1 et al.) in an in vitro setting. Employing RT-qPCR, they quantified the expression of genes related to MHC-antigen processing and presentation (MHC-APP), identifying an upsurge in MHC-APP gene expression in cells treated with 3-NPA. Further analysis showed CII dysfunction-induced nuclear MHC-APP gene expression is independent of interferon signaling and partly driven by NLRC5 transcriptional responses.

Previous research showed that succinate, regulated by CII, impacts nuclear gene expression. The researchers found that inhibiting CII caused succinate to accumulate, influencing MHC-APP gene expression. They presented four key findings: 1) Cells with inhibited CII had elevated succinate levels; 2) Adding cell-permeable succinate to these cells increased MHC-I and MHC-APP gene expression; 3) Reducing glutamine in CII-impaired cells decreased both 3-NPA-induced succinate buildup and MHC-I/MHC-APP gene expression in YUMM1.7 cells; 4) The silencing of OGDH in CII-deficient cells markedly decreased both succinate levels and MHC-I expression. Tumor cells often avoid immune detection by downregulating MHC-APP expression or becoming IFN-γ resistant. In this context, the study’s findings are crucial. They showed that CII inhibition in mitochondria led to increased succinate, which then enhanced transcription and antigen presentation of MHC-APP genes, improving tumor immunogenicity.

Subsequently, the authors demonstrated that succinate increased MHC-APP transcription through the inhibition of histone demethylases and the modulation of tumor epigenetics. They confirmed that trimethylation of histone H3 at lysine 4 (H3K4me3) and H3K36me3 are pivotal markers regulating antigen presentation in response to accumulated succinate. Additionally, the build-up of succinate, consequent to the inhibition of CII, modified the epigenetic profile of MHC-APP genes. This modification transpired through the inhibition of the enzymes histone demethylases KDM4 and KDM5, coupled with the elevation of NLRC5 levels, collaboratively prompting the transcription of MHC-APP genes.

Nonetheless, the systemic inhibition of CII as a strategy to increase succinate levels in tumors may not be viable due to its associated neurotoxicity. This could potentially initiate new tumor formation and likely cause other detrimental physiological impacts on healthy cells and tissues [4]. The Methylation Control J protein (MCJ) is a naturally occurring protein that interacts with CI, situated in the mitochondrial inner membrane. Knockdown of MCJ resulted in higher CI activity compared to CII and led to the formation of mitochondrial supercomplexes [5]. Building on this, *Kailash* and colleagues re-established the ETC by knocking down MCJ (thereby decreasing CII activity and facilitating preferential electron entry into mitochondria via CI). They ultimately demonstrated that this reconnection of the ETC could yield a potent antitumor response without affecting mitochondrial respiration in noncancerous cells. These findings propose a novel approach to enhance tumor immunogenicity by modulating the ETC. In summary, their findings demonstrated that tumor proliferation can be impeded by augmenting succinate and MHC-I levels within tumor cells, achievable either through *Mcj*-Knockout or direct CII inhibition. This increase in levels bolstered the immunogenicity of tumor cells and facilitated the activation and infiltration of tumor-reactive effector CD8+ T cells, as illustrated in Fig. 1.

**Fig. 1:**
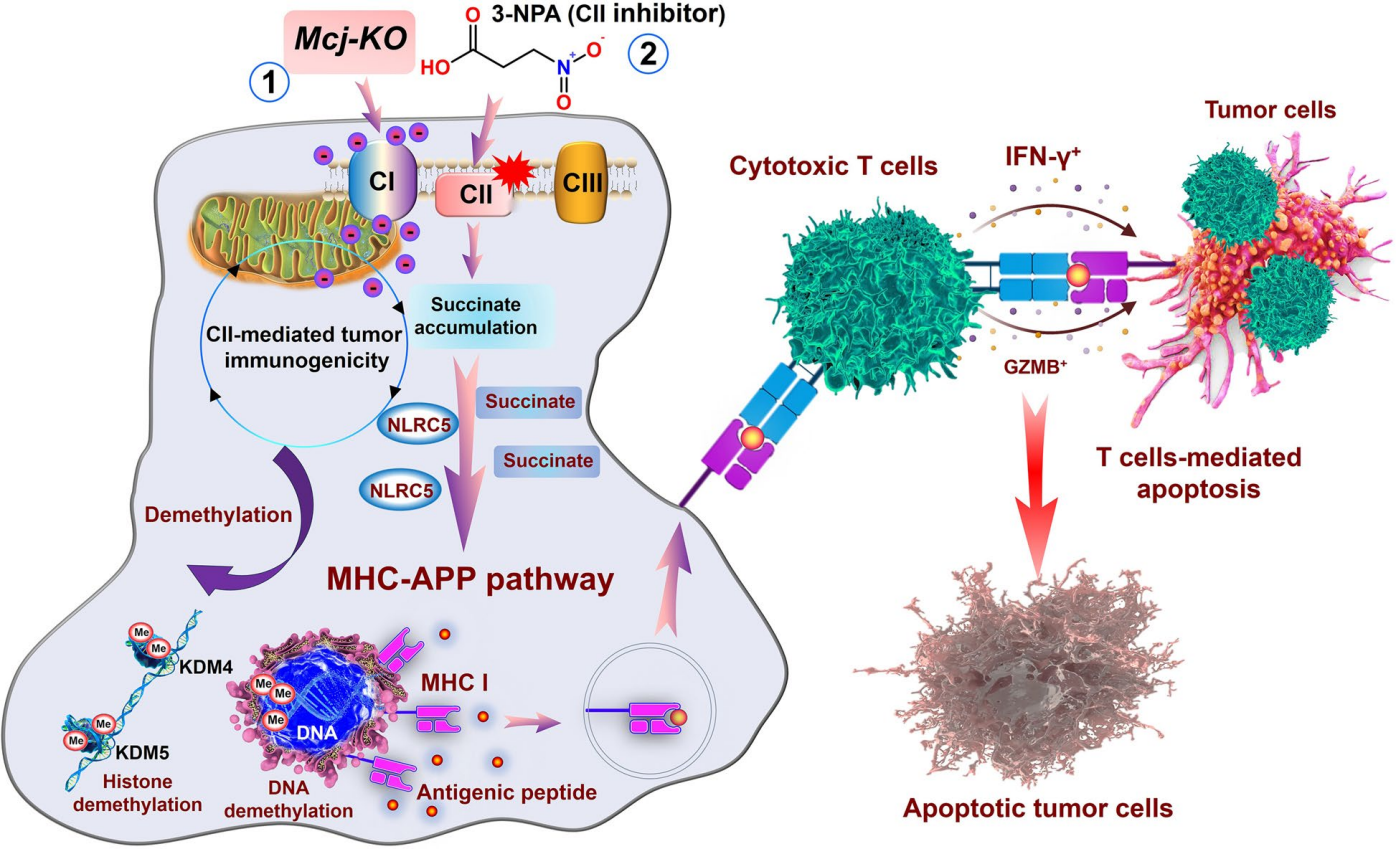
Schematic illustration of CII-mediated tumor immunogenicity. It outlines two methods: *Mcj*-KO and CII activity inhibition, both increasing succinate and changing the epigenetics of MHC-APP genes. This includes suppressing KDM4 and KDM5, raising NLRC5 levels, and boosting MHC-APP gene transcription. Using 3-Nitropropionic Acid enhances MHC-APP gene expression and antigen presentation in tumors, leading to higher IFN-γ and GZMB production by T cells, targeting tumors effectively

In this research, the team systematically investigated the role of CII in regulating tumor immunogenicity. Their findings revealed that CII functional impairments attenuated melanoma growth, primarily by augmenting antigen presentation and T cell-mediated cytotoxicity. This effect was mediated through the succinate-regulated transcriptional and epigenetic activation of the MHC-APP gene, independently of interferon signaling pathways. Furthermore, they demonstrated that silencing the MCJ protein, thereby facilitating preferential electron flow through CI, reconnects the ETC and induces an antitumor response. Significantly, this approach avoids the typical side effects associated with a widespread decrease in mitochondrial respiration in non-cancerous cells. Their results highlight a potential therapeutic approach for enhancing tumor immunity by modulating the ETC, underpinned by the pivotal roles of succinate and MHC-I in tumor immunogenicity. This approach offers a novel theoretical framework and therapeutic avenue for transforming “cold tumors” into “hot tumors,” thereby augmenting the efficacy of antitumor therapies.

Based on these insights, several promising research avenues emerge:

1. Expanding the scope beyond melanoma to other tumor types, to ascertain the broader implications of CII functional defects and their differential impact on immune responses.
2. Investigating the influence of CII impairments on immune cells, beyond their role in tumor cell antigen presentation, to gain a holistic understanding of their effect on the tumor immune landscape.
3. Exploring the potential synergistic effects of combining CII functional defect interventions with immune checkpoint inhibitors to amplify tumor immune responses and enhance therapeutic outcomes.
4. Evaluating the dual therapeutic potential of integrating gene editing techniques with mRNA vaccine strategies.
5. Probing the relationship between CII functional defects and tumor immune evasion, a critical factor in tumor progression and resistance to treatment.

## Data Availability

Not applicable.
